# The ongoing quest for the independent patient: an interview study on healthcare professionals’ perspectives on integrating self-monitoring of blood pressure in hypertension care

**DOI:** 10.1186/s12875-026-03244-2

**Published:** 2026-03-05

**Authors:** Erica Kelemit, Elnura Halmambetova, Evalill Nilsson, Cecilia Fagerström, Linda Ljungholm

**Affiliations:** 1 Department of Medicine and Optometry, Faculty of Health and Life Sciences, Linnaeus University, Kalmar-Växjö, Sweden; 2 Department of Research, Region Kalmar County, Kalmar, Sweden; 3 Department of Health and Caring Sciences, Faculty of Health and Life Sciences, Linnaeus University, Kalmar, SE-392 31 Sweden

**Keywords:** Hypertension, Self-monitoring, Person-centred care, Patient-reported data, Self-care, E-health

## Abstract

**Background:**

Home-based self-monitoring of blood pressure has been proposed as a way to enhance patient engagement and self-management of hypertension while potentially reducing pressure on primary healthcare services. Although the effects of self-monitoring are well-documented, less is known about healthcare professionals’ experiences of supporting patients with self-monitoring, or the potential added value of patient-reported measures (questionnaires) of health and self-care in hypertension care.

**Aim:**

To explore healthcare professionals’ experiences of supporting patients in self-monitoring of blood pressure and, in addition, their reflections on the idea that patient-reported measures of health and self-care could provide added value in hypertension care.

**Method:**

A qualitative study with an inductive approach. Data were collected through semi-structured interviews with 15 informants involved in the care of patients with hypertension who self-monitored their blood pressure. The interviews underwent reflexive thematic analysis.

**Results:**

Healthcare professionals’ experiences of supporting patients with self-monitoring of blood pressure suggest that self-monitoring can promote patient independence through participation in own health and self-care. However, it may also trigger anxiety in some individuals, underscoring the need for appropriate education and support. When presented with the idea of complementing self-monitoring of blood pressure with questionnaires, healthcare professionals reflected on potential benefits, such as enabling individualised care, identifying patients with greater need for support, and facilitating more preventive approaches to hypertension and its complications.

**Conclusion:**

Self-monitoring of blood pressure can support hypertension self-management, particularly when data reporting is simplified. Combining the practices of self-monitoring of blood pressure with patient-reported measures may have the potential to enhance person-centred and preventive care. However, clear responsibilities and standardised procedures and professional support are essential to balance patient autonomy with safety.

**Supplementary Information:**

The online version contains supplementary material available at 10.1186/s12875-026-03244-2.

## Background

Hypertension, or high blood pressure, is a leading risk factor for serious cardiovascular diseases and represents the single greatest cause of morbidity and mortality globally [1]. According to the World Health Organization (WHO), about a third of the adult population aged 30–79 years worldwide is affected by hypertension [2]. This contributes to increased demands on healthcare services and, in combination with limited healthcare resources, necessitates the development of new strategies for managing patients with chronic conditions. Enhanced self-care, including self-monitoring, is regarded as one such strategy [3]. The WHO defines self-care as an individual’s ability to manage their own health conditions, with or without support from healthcare professionals (HCPs) [4]. Patients who are more actively engaged in their self-care generally experience better health outcomes and report higher health-related quality of life [5]. This may contribute to reduced healthcare costs. However, patients often struggle to integrate self-care into their daily routines, which underscores the need for HCPs to educate, support, and follow up.

In the context of hypertension, self-care encompasses three key components: lifestyle modifications, pharmacological treatment, and self-monitoring of health-related factors. Lifestyle changes, such as adopting a healthy diet, engaging in regular physical activity, and reducing stress [6], are typically the first line of treatment following diagnosis and are recommended at all stages of hypertension. Nevertheless, adherence to these changes is often low, as is adherence to prescribed medication [7, 8]. The reasons for this are multifaceted and may relate to economic factors, treatment complexity, disease characteristics, individual circumstances, or structural aspects of the healthcare system [9]. Given that hypertension is frequently asymptomatic, the absence of noticeable positive physical effects from treatment may lead to poor adherence [9, 10]. Furthermore, antihypertensive medications may cause side effects that negatively impact quality of life, which can be particularly difficult for patients to accept when the condition itself often does not produce obvious symptoms.

Self-monitoring involves the regular measurement of health-related factors associated with a specific condition, often using dedicated equipment [11]. In the case of hypertension, this entails patients measuring their own blood pressure using a blood pressure measurer (BPM) outside of clinical settings, such as at home, and reporting the readings to healthcare providers [6]. Hereafter, self-monitoring of blood pressure will be referred to as SMB.

Other digital tools that may support healthcare delivery include patient-reported measures (PRM) regarding health and self-care (e.g., patient-reported outcome measures, patient-reported experience measures, and patient activation measures) in the form of electronic questionnaires; hereafter, collectively referred to as PRMs. Typically, such measures are validated for use within or sometimes across diverse patient populations and clinical settings but seldom integrated into electronic health record systems (EHR) [12]. As SMB continues to evolve though its effects on blood pressure reduction, whereas increased patient involvement remains to be established, further follow-up and research are needed. Furthermore, though PRMs are increasingly used in research, they are still rarely integrated into routine care. Although there are hypertension-specific PRMs, such as the Self-care of Hypertension Inventory [13], no Swedish version has so far been validated in a primary care SMB context for mild and moderate essential hypertension. Therefore, the aim of the present study is to describe HCPs’ experiences of supporting patients in SMB and explore their perspectives on the idea of a potential added value of PRMs in hypertension care.

## Method

### Design

The study is part of a larger project (FORSS-984272) conducted in southeastern Sweden during 2023–2024, aimed at exploring PRMs and SMB from various perspectives. In addition to the present sub-study, the main project also included, among other things, an interview study on patient perspectives [14].

The present study used a qualitative design consisting of two rounds of interviews (A and B). The first (A) was conducted before the patient interviews and focused on the HCPs’ experiences of SMB. Since the patient interviews came to include the aspect of PRM in addition to SMB, it was decided to add more interviews with HCPs to cover that perspective as well. Initially, the intention was to produce two separate articles; however, the narratives provided by the informants in the two rounds turned out to be interrelated and mutually reinforcing, and after reading all the transcripts the decision was made to merge the two interview rounds. This was also reinforced by the fact that participants in Round B discussed the hypothetical value of PRM in general terms, not in relation to any specific questionnaire or its actual use, and the data material was therefore in the end not considered to need its own article. The data underwent reflexive thematic analysis as described by Braun and Clarke [15].

### Setting

In southeastern Sweden, efforts are underway to establish routines that enable patients to self-monitor blood pressure using either their own BPM or one borrowed from their primary care centre. Patients are expected to follow the provided instructions and guidelines for measuring blood pressure. If using their own BPM, calibration can be performed at a primary care centre or pharmacy. BPMs borrowed from primary care centres are calibrated regularly. Patients can report their readings digitally, on paper, or verbally during visits to the primary care centre. Typically, follow-up occurs after one month, six months, and then annually. For patients who have changed medication, follow-up takes place within four to six weeks. Feedback and review of readings are handled by a nurse unless deviations are noted, in which case the physician is informed. Currently, no supportive tools such as PRMs are used in this context.

### Participants

A strategically guided purposive sampling was employed to recruit HCPs in the southeastern region based on their experience of supporting SMB patients and their willingness to participate. To ensure variation in the data, the participant group represented different professional roles, with varying ages and years of work experience (Table 1).

**Table 1:** Characteristics of the study participants in the two rounds A and B

Description of interviews	Type of interview	Profession and number of participants (*n*)	Gender (*n*)	Interview mode (*n*)	Interview length in minutes (range)
A. Perspectives on SMB in hypertension care	Group interview	Unit manager (1) Nurse (2)	Man (1) Woman (6)	Online (3) On-site (1)	32 (30–34)
	Paired interview	District nurse (2)			
	Individual interviews	Physician (1) Unit manager (1)			
B. Perspectives on SMB and the added value of PRMs in hypertension care	Individual interviews	District nurse (3) Nurse (3) Physician (2)	Woman (8)	Phone (1) Online (7)	44 (38–60)

Participants for interview round A were recruited through email invitations sent to managers at primary care centres in southeastern Sweden known to have initiated SMB, as well as to two private hypertension centres operating in the region. Participants for round B were recruited via email invitations sent to managers at primary care centres. No participant participated in both rounds.

### Data collection

Interviews in round A lasted an average of 32 min and included one group interview conducted online with a unit manager and two nurses, one paired interview conducted on-site with two district nurses, and two individual interviews (Table 1). The mix of interview modes was partly due to time constraints and partly because the staff expressed a wish to be interviewed together to complement each other and provide a more comprehensive picture. These interviews explored HCPs’ experiences of supporting patients in SMB, including processes and routines regarding patients with newly diagnosed hypertension. Round B lasted an average of 44 min and consisted of eight individual interviews conducted by phone or online (Table 1). Besides exploring HCPs’ experiences of supporting patients with SMB, interviews in round B also included HCPs’ views on the hypothetical potential added value of PRMs in relation to other available health data. The overarching thematic areas in the interview guides (which were mainly similar except for PRM) included patient involvement in care, routines for SMB and follow-up, and, in the guide used in Round B, attitudes toward PRMs. All interviews were conducted by three members of the main project group (MB, ME, HH).

### Data analysis

Data were analysed using reflexive thematic analysis according to Braun and Clarke’s six-phase method [15]. A reflexive stance was adopted throughout the analytical process, recognizing that themes are not passively discovered in the data but are actively and interpretively developed by the researchers. The analysis was iterative, involving continuous movement between data, codes, and emerging interpretations, as well as ongoing consideration of how clinical experiences and the disciplinary backgrounds of the research team shaped the analytical outcomes.

In the initial phase, all interviews were read repeatedly by EK, EH to gain an overall understanding of the material and to reflect on early assumptions and analytical expectations. In the second phase, meaning-bearing units were identified and coded, where codes were treated as provisional analytical tools rather than fixed descriptive labels. Coding involved an interpretive engagement with the data, where their essence was captured in a single idea. During the third phase, EK, EH, LL, EN began to construct a preliminary theme by exploring meaning patterns across different code clusters. Subthemes were used to capture different dimensions of these early interpretations. In the fourth phase, the developed theme and its subthemes were reviewed through a reflexive return to all coded excerpts and the entire dataset. This step involved an analytical dialogue between all researchers, which allowed for challenging, refining and deepening interpretations through contrasting perspectives from the researchers’ different perspectives in medicine, nursing, health sciences and health informatics. In the fifth phase, the theme was further defined and delimited. The naming of themes was guided by the central concept that best captured the underlying meaning and analytical narrative conveyed by the data. The main theme, “The ongoing quest for an independent patient,” was developed to reflect how HCPs negotiated and reflected on their roles and expectations when supporting SMB patients. All researchers contributed to this step to broaden the reflexive breadth of the analysis. In the sixth and final phase, the analytical narrative was consolidated by writing as a further interpretive process where the outlines and meaning of the themes were further refined. Both datasets (A and B) contributed relevant information to the purpose of the study.

## Results

**Fig. 1:**
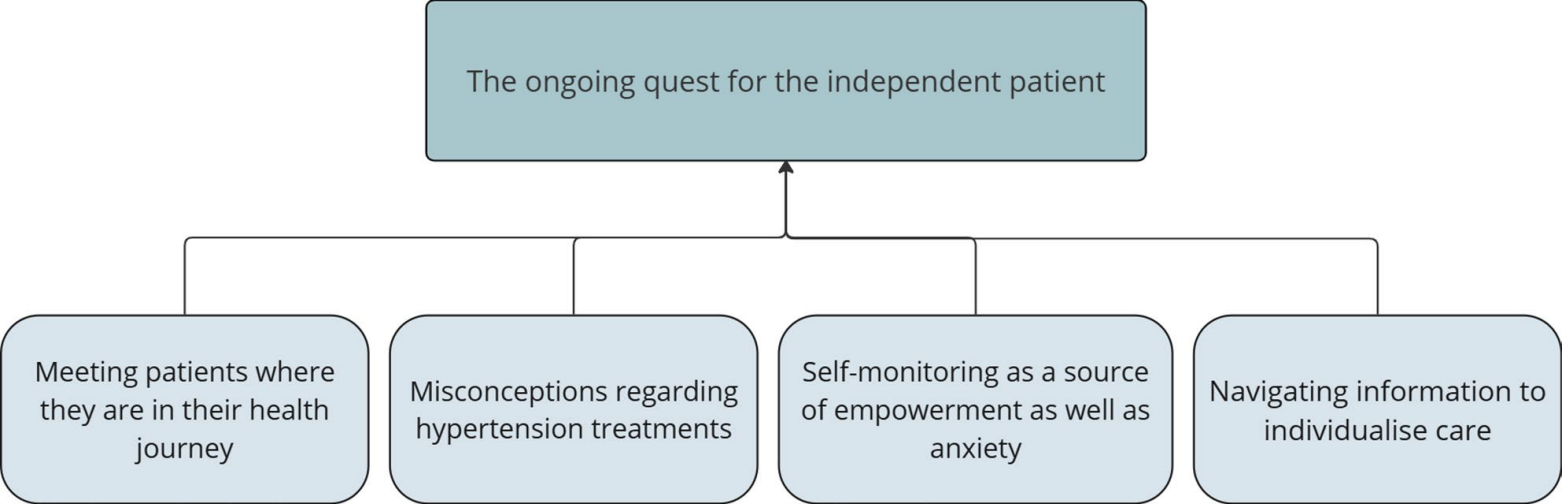
Description of the theme and subthemes

One main theme and four subthemes were generated to capture HCPs’ experiences and perspectives of supporting patients in SMB and the potential added value of PRMs (see Fig. 1).

### The ongoing quest for the independent patient

HCPs’ experiences of supporting patients in SMB and their perspectives on the potential added value of using PRMs consistently emphasised the promotion of patient autonomy, independence, and self-care. By guiding and informing patients about medication, promoting lifestyle changes, and encouraging SMB, HCPs sought to support patients in taking responsibility for medication adherence, adopting healthier lifestyle habits, and engaging in SMB. Although HCPs perceived that some patients responded positively to increased responsibility, they also highlighted challenges in promoting patient independence. Alongside SMB, PRMs – particularly if they were to be integrated into the EHR – were perceived as potentially valuable tools for tailoring care to individual needs and identifying patients who required additional support for hypertension self-management, thereby contributing to the prevention of hypertension and its related complications.

### Meeting patients where they are in their health journey

Despite HCPs’ attempts to encourage a healthy lifestyle, for instance through increasing physical activity, adjusting diet, or managing stress, they reported that many patients struggled to translate this knowledge into action. According to HCPs, these difficulties persisted despite patients’ SMB and awareness of how lifestyle affects blood pressure.

HCPs employed several strategies to support motivation, such as discussing personal goals and following up on agreed self-care plans, including exercise and dietary changes. Despite these efforts, long-term change was perceived to require resources and support rarely available in primary care. According to HCPs, primary care followed the principle ‘a lot of care for the few, less care for the many’ by prioritising patients with uncontrolled hypertension. Patients with stable blood pressure were mainly supported by nurses and saw physicians only when values deviated, typically attending follow-ups every one to two years. Patients who required intensive support were identified during annual checkups. In line with this, HCPs underlined that greater patient responsibility could help ease pressure on primary care personnel. They noted that their work felt more fulfilling when patients learned to manage their disease and conversely felt like a failure when patients were not receptive to information.

HCPs expressed different views on PRMs. Some perceived little value in their use, citing time constraints or a preference for relying on clinical experience to assess patients’ situations. Others suggested that PRMs could aid in preventing hypertension and its complications while offering valuable insights into each patient’s unique needs and preferences, thereby enabling early differentiation of patients.

We’re so into helping the ill now, those who already have high blood pressure, we treat them, of course, but how do we prevent it from happening? How do we reach the prehypertensive patients? That’s where I see a huge gain in PRMs. [Participant B2]

PRMs can make the care more person-based, because then there is a baseline to start from. [Participant B5]

Overall, participants noted that patients seemed to appreciate receiving coaching support. SMB and PRMs were described as potentially helpful tools in supporting lifestyle changes by clarifying the purpose of self-care and making progress more tangible.

[With SMB, ] patients can see the relationship between the choices and changes they make and the effects on their blood pressure, and patients often want proof that what they are doing is right. [Participant B6]

I think patients may be more motivated when using PRMs. [Participant B6]

Some clinics offered a ‘hypertension school’, where patients received education about the disease. However, these sessions were irregular and dependent on the number of patients enrolled, which led to them often being cancelled. Some respondents reflected on whether the possibility of digital participation could increase enrolment, as all patients in the region would then be able to participate in the sessions. In support of this notion was the statement by a respondent representing a clinic which operated wholly digitally. They said that the clinic offered a variety of different digital programmes to promote lifestyle changes, which were appreciated by both healthcare providers and patients.

### Misconceptions regarding hypertension treatment

Informing and discussing medication with patients was mentioned as one of the main responsibilities of physicians and nurses. Patients, in turn, were expected to adhere to the chosen treatment and contact their provider if they had any concerns. A follow-up was typically conducted three to four weeks after initiating medication, to evaluate effectiveness. One respondent noted:

We are quite willing to change medications if the patient wants to, as medication adherence is central to good treatment. [Participant A2]

Some HCPs noted that adherence to antihypertensive medication was not always feasible among patients with financial constraints, leading to unintentional non-adherence. However, most participants underscored that patients lacked an understanding of the treatment goals and the chronic nature of the condition, citing this as a more common reason for poor adherence. For example, some patients took medication only for a short time, discontinuing use without consulting healthcare when they felt symptom-free or reached their target values.

Some patients think they should only take the medicine until their blood pressure is normal, and then they stop. [Participant B2]

As patients were responsible for notifying HCPs when their medication ran out, tracking patients who discontinued medication was challenging; there was no system for flagging non-renewals. According to the HCPs, this often led to them losing track, with discontinuation typically detected only at annual follow-ups. Several experiences indicated that some patients adjusted their dosage themselves, without consulting HCPs. This was due to fear of side effects, previous negative experiences, or the belief that medication was no longer needed when blood pressure values improved. In such cases, HCPs felt that building trust and open dialogues was crucial for promoting adherence and preventing risky decisions. According to the participants, PRMs could potentially be useful in identifying whether patients had discontinued or altered their medication on their own. This could enable targeted communication about the risks, for instance of heart failure or stroke.

### Self-monitoring as a source of empowerment as well as anxiety

According to HCPs, encouraging patients to SMB was essential for engaging them in their own care. It was described as a means to ‘govern oneself’, thereby facilitating self-efficacy. Patients were instructed to either purchase a BPM or borrow one from the clinic. After a patient was diagnosed with hypertension, most participants recommended them to conduct SMB once a month to assess whether their blood pressure was within target values. Patients were also instructed to self-monitor directly after the initiation of medication, during dose and medication adjustments, and prior to follow-up appointments, including annual check-ups. Overall, SMB was perceived as a way to reduce the need for clinical visits and to free up time for other tasks in primary care:

We had so many blood pressure appointments that we didn’t have time for other care. [Participant B8]

At the same time, HCPs experienced that SMB posed challenges for some patients such as older adults, individuals with cognitive impairments, or those with limited health and digital literacy. Participants also noted that SMB could induce stress in patients who were prone to worry. These patients often became anxious when confronted with even normal daily fluctuations in readings. In some cases, having access to their own device led patients to become somewhat obsessed with measuring their blood pressure.

They measure every day and panic as soon as there is a slight increase. [Participant A1]

Some are very control-oriented and anxious. In those cases, having their own BPM isn’t good. [Participant B2]

Most HCPs stated that patients often worried about elevated values rather than being observant of trends in the values.

We can’t sit and monitor the patients; they need to be educated on when to react to their blood pressure values. [Participant B8]

In these cases, some healthcare professionals instead recommended 24-hour automated blood pressure monitoring to reduce anxiety and help patients recognise how stress affected blood pressure and the importance of relaxation.

Therefore, SMB was experienced as not only helping patients take more responsibility for their health, but also as requiring greater support, including meaningful feedback and assistance in interpreting results through written information, colour-coded guidelines, or digital tools. HCPs suggested that enabling for patients to follow trends over time in a simple format could support self-management of hypertension.

### Navigating information to individualise care

To provide optimal care for each individual patient, patient resources, circumstances, and clinical information, including laboratory tests (e.g., kidney and liver function), self-monitored blood pressure values, and reported symptoms were all taken into account to guide medical decisions and tailor care. Some participants thought that combining SMB with PRMs could be a way to further individualise care. However, several HCPs described instances in which clinical deterioration was identified too late due to uncertainty about who was responsible for registering the patients’ self-reported blood pressure values in the EHR and providing feedback on the reported data. Similar concerns were raised regarding PRM data.

We don’t always know who’s responsible for looking at those questionnaires. [Participant B6]

Some physicians emphasised the importance of collaboration between nurses and physicians, suggesting that nurses should take primary responsibility for handling self-reported blood pressure values and PRM data, thereby enabling physicians to make informed clinical decisions, such as adjusting medication. At the same time, several nurses noted the need for physicians to communicate more clearly with patients and to place greater responsibility on patients for self-management of hypertension.

In the absence of clear routines and defined areas of responsibility for handling patient-reported blood pressure values, HCPs developed their own strategies to manage the limitations of the existing systems. For example, self-reported blood pressure values submitted by patients via SMS, email, or handwritten notes were manually rewritten or pasted into the EHR. Most participants underscored the need to streamline workflows for managing patients with hypertension, to free up time for other clinical tasks. One frequently suggested improvement was the digitalisation of blood pressure reporting, including automated alerts for HCPs in cases of abnormal values. Such systems were anticipated to facilitate follow-up care by allowing easy access to a comprehensive overview of patients’ blood pressure readings.

Paper-based reporting can be difficult; it would be easier digitally. [Participant B7]

Some noted that SMB and digital PRMs might be more beneficial for younger patients, who were generally perceived as ‘more willing to take responsibility for their health’, whereas ‘reskilling’ older patients was considered more challenging.

I think there are a lot of different patient groups, for some it’s great that they can manage on their own and can report their values to us from time to time, while there are also groups that can’t cope with the digital world, who are multi-morbid and fragile. They need to be taken care of in a different way. So, if there is more digitalisation, more efficiency, then we have the resources to take care of those who really need to be taken care of. [Participant B4]

Given the overall difficulty in engaging patients, HCPs suggested that PRM data reports should be visually appealing to capture patients’ attention, and they also recommended that such data be collected in conjunction with annual check-ups.

## Discussion

Pursuing patient independence emerged as a central theme, with SMB – alongside lifestyle modification and medication adherence – seen as key to improving hypertension self-management, supporting preventive care, and ultimately reducing primary care workload.

Furthermore, this study suggests that PRMs could help HCPs tailor self-management support to patients’ individual needs if, as anticipated, PRMs enable more targeted communication and contribute to the prevention of hypertension and its complications. However, to fully realise the potential of PRMs and SMB, clear routines, well-defined areas of responsibility, and streamlined workflows need to be developed in hypertension care.

HCPs in this study who had integrated SMB into daily practice recommended SMB as a strategy to enhance patient participation. This finding echoes those of the main project’s sub-study on patients’ perspectives, which showed that SMB and PRMs have the potential to empower patients to engage more actively in symptom management and self-care [5, 16, 17].

The present study also suggests that SMB may provoke stress and anxiety in patients, a side effect that is well-documented in the literature [18, 19] (Halmambetova E, Nilsson E, Fagerström C, Årestedt K, Bjöersdorff M, Ljungholm L: Self-monitoring of blood pressure and self-assessment of self-care: an interview study among patients with hypertension, submitted). Such anxiety is often attributed to panic over ‘bad’ readings, constant reminders of the illness, and a lack of confidence in interpreting results [20]. Conflicting information from different HCPs regarding target values, combined with difficulties in assessing online health information, may further contribute to patient uncertainty [19].

HCPs further reported that some patients independently adjusted or discontinued their medication based on SMB values. Although this may reflect increased engagement, it raises concerns about medication safety in the absence of structured guidance. A systematic review on SMB in patients with hypertension similarly highlights the risks of unsupervised medication changes and underlines the importance of shared decision-making and continuous professional feedback when promoting SMB [17].

When HCP perspectives are compared with patient perspectives, a divergence in approaches to SMB becomes apparent [21] Patients tend to link blood pressure fluctuations to lifestyle factors, particularly stress, and view SMB values as closely connected to daily behaviours [20] (Halmambetova E, Nilsson E, Fagerström C, Årestedt K, Bjöersdorff M, Ljungholm L: Self-monitoring of blood pressure and self-assessment of self-care: an interview study among patients with hypertension, submitted). In contrast, HCPs in the current study appeared to emphasise SMB as a means to foster independence and manage workloads in primary care. This reflects broader tensions in the literature, where self-management is increasingly promoted in resource-constrained primary healthcare systems [22]. To fully realise the potential of SMB, future implementation strategies should integrate person-centred support, structured feedback, and explicit attention to lifestyle counselling, ensuring that patient autonomy is supported rather than assumed.

The issues of lifestyle changes [7, 9, 21, 23, 24] and non-adherence to prescribed medication [8–10, 18, 25–27] are well-documented. According to the literature, medical information risks becoming impersonal [9], and limited resources often lead to standardised encounters, with little time for person-centred care [28]. Although HCPs in the present study recognised this issue and actively sought to promote patient understanding and engagement, lifestyle changes and adherence to medication were not always achieved. Studies on patient activation suggest that some patients may not be receptive to information if they are in the early, passive stages of activation. At this stage, patients tend to be unprepared, which can limit their ability to process information – even when it is well-communicated [29]. This may help explain why, despite employing multiple strategies to educate patients about hypertension, HCPs find that patients struggle to process and internalise information. In recent decades, healthcare practice has shifted from a model of medication compliance, where patients were expected to follow recommendations passively, to a model of adherence, which recognises patients as active partners in jointly agreed-upon decisions. More recently, the concept of concordance has emerged, emphasising a co-creative partnership in which patients and HCPs share responsibility in a balanced way [30]. Within this evolving paradigm, another challenge lies in assessing patients’ levels of health literacy to tailor consultations appropriately. Health literacy encompasses both individual-level factors (e.g., knowledge and psychological resources) and system-level factors (e.g., healthcare structures and stakeholder responsibilities), underscoring the need to address both domains. A recent review found that HCPs often have limited knowledge of the concept of health literacy but express a willingness to learn more about it [31].

Given these multifactorial challenges involved in promoting patient independence and autonomy in hypertension care, the question arises of if PRMs – as a complement to SMB – could facilitate more effective support, including the identification of patients who have discontinued their medication. The HCPs in the present study viewed PRMs as a means to support more tailored person-centred care, a perspective supported by the main project’s patient study, which suggested that PRMs could encourage reflection, preparation, and more productive consultations [32]. Conversely, when PRMs are collected but not meaningfully integrated into care, patients may experience frustration or perceive the process as burdensome [33]. Other studies report that completing PRMs helps patients articulate issues that might otherwise be overlooked during consultations, particularly in time-constrained clinical settings [34, 35].

Although HCPs recognised the potential benefits of SMB and PRMs, they also noted that routines and workflows for integrating and managing such data are not yet fully established in clinical practice. One point that was not explicitly raised in the current study but has been shown in previous research is that SMB may generate substantial amounts of blood pressure data, not all of which are likely to be clinically meaningful [36]. In addition, manual data entry may increase staff workload. Standardised reporting systems and automated data transfer and analysis strategies could help address these challenges [17, 19, 36]. Without such systems, the integration of digital data risks becoming fragmented and data may be underutilised. Further research should explore how PRMs can be effectively implemented in primary care as a complement to SMB. Studies could also examine whether SMB combined with educational interventions and appropriate support can reduce anxiety and improve long-term quality of life among stress-prone patients. Although the HCPs in the present study supported empowering patients to gain greater control of their health through SMB, questions remain about the optimal measurement frequency and for which patient groups SMB is most effective.

### Strengths and limitations

The diverse professional backgrounds (i.e., specialist nurses, district nurses, clinic managers, and physicians) of the HCPs, combined with the consistency in their views on SMB and PRM, suggest that the findings may be transferable to similar contexts. The intention was to include both men and women, but few men in the region were working with SMB at the time and therefore the study was unable to include more than a single man. While this reflects the gender dynamics within Swedish primary care, it could potentially limit transferability. Participants provided a balanced perspective on the subject, highlighting both positive and negative aspects of SMB and PRMs, which enhances the credibility of the study. Including different types of interviews can affect consistency, but at the same time, group interviews can serve as a complement to individual interviews, as the group creates interaction between the participants, which contributes to reflection.

Although guidelines for hypertension care are largely uniform throughout Sweden, variations in SMB processes across regions and countries may affect transferability. To increase transferability, the study context has been described carefully [37]. However, transferability limitations should always be considered.

The credibility of this study was enhanced through researcher triangulation, as the research team comprised members from four distinct disciplines. To minimise researcher bias, the researchers continuously discussed and reflected on their pre-understandings throughout the analysis process.

In line with Braun’s and Clarke’s conceptualisation of data saturation, the study prioritised data richness over traditional saturation, considering the sample adequate once sufficient depth and diversity to develop meaningful and well-supported themes were achieved [38]. Quotations were included throughout the findings to illustrate participants’ perspectives and support the transparency of the analysis [39].

Another consideration is that participants reflected on anticipated rather than direct experiences of hypertension-specific PRMs, as such tools were not used in hypertension care at the time of the interviews. However, the HCPs’ extensive professional experience and familiarity with PRMs for other health conditions likely enabled them to envision their potential use.

## Conclusion

SMB can support patients in managing hypertension. To further enhance the benefits of SMB, the reporting of self-measured blood pressure values should be simplified and automated. The ability of HCPs to interpret SMB data in combination with information from PRMs might contribute to more individualised and person-centred care. The added value of PRMs lies in their potential to increase the capacity of HCPs to deliver such care, thereby creating opportunities for more preventive approaches to hypertension and its complications.

The study highlights various components of hypertension care – including medication adherence, lifestyle modifications, and SMB – that can promote patient independence. However, these approaches also involve risks if patients lack adequate guidance and support.

To fully realise the benefits of SMB and PRMs, standardised procedures and clearly defined responsibilities for data management are required. Overall, the study underscores the ongoing need for HCPs to balance the promotion of patient independence with the provision of appropriate support in hypertension care.

## Supplementary Information

Supplementary Material 1.

## Data Availability

The datasets analysed during the present study are not publicly available due to privacy or ethical restrictions, in line with the approval of the Swedish Ethical Review Authority.

## Abbreviations

BPM: Blood pressure measurer
EHR: Electronic health record system
HCP: Healthcare professionals
PRM: Patient reported measures
SMB: Self–monitoring of blood pressure
WHO: World health organization
