# Supplementary material for: The ongoing quest for the independent patient: an interview study on healthcare professionals’ perspectives on integrating self-monitoring of blood pressure in hypertension care
Source: BMC Prim Care. 2026 Mar 5;27:126. doi: 10.1186/s12875-026-03244-2 (PMC13063534; doi:10.1186/s12875-026-03244-2)
Supplement: Supplementary file 1 — Supplementary Material 1. [file 12875_2026_3244_MOESM1_ESM.docx]

# The ongoing quest for the independent patient: an interview study on healthcare professionals’ perspectives on integrating self-monitoring of blood pressure and self-assessment of health and self-care in hypertension management

Erica Kelemit^2^, Elnura Halmambetova¹, Evalill Nilsson¹, ², Cecilia Fagerström¹, ^3^ & Linda Ljungholm^3^

¹Department of Research, Region Kalmar County, Kalmar, Sweden

²Department of Medicine and Optometry, Faculty of Health and Life Sciences, Linnaeus University, Kalmar-Växjö, Sweden

^3^Department of Health and Caring Sciences, Faculty of Health and Life Sciences, Linnaeus University, Kalmar-Växjö, Sweden

ORCID ID

Linda Ljungholm: 0000-0003-0338-7610

Evalill Nilsson: 0000-0002-4497-8313

Cecilia Fagerström: 0000-0002-4257-282x

Corresponding author

Linda Ljungholm

Faculty of Health and Life Sciences, Linnaeus University

SE-392 31 Kalmar, Sweden

E-mail: [linda.ljungholm@lnu.se](mailto:linda.ljungholm@lnu.se)
